# Types and frequency of ovarian masses in children over a 10-year period 

**Published:** 2015

**Authors:** Naser Sadeghian, Irandokht Sadeghian, Alireza Mirshemirani, Ahmad Khaleghnejad Tabari, Javad Ghoroubi, Fatemeh Abdollah Gorji, Fatollah Roushanzamir

**Affiliations:** 1 Pediatric Surgery Research Center, Shahid Beheshti University of Medical Sciences, Tehran Iran.; 2 Clinical Research Development Center, Mofid Children's Hospital, Shahid Beheshti University of Medical Sciences, Tehran Iran.

**Keywords:** Ovarian tumors, Abdominal pain, Children, Postoperative outcome

## Abstract

**Background:**

Ovarian masses represent a range of pathology from benign cyst to highly aggressive malignant tumors. It has been estimated that gynecologic malignancy account for approximately 2% of all types of cancer in children, 60-70% of these lesions arise in the ovary.

**Methods:**

All ovarian masses which were resected or biopsied in Mofid Children's Hospital from 2002 to 2012 were reviewed retrospectively. Patient’s age, presenting symptoms, surgical procedures, pathological diagnosis, postoperative treatment, and outcome were obtained from medical records.

**Results:**

Fifty-seven girls (aged 40.2±57months with the range of 1 day to 15 years) underwent different types of ovarian operations (24 salpingo-oophorectomies, 10 oophorectomies, 21 ovarian cystectomies, and 2 ovarian biopsies). 50 children had unilateral ovarian mass (49.1% right and 38.6 left, respectively). The most common presenting symptoms were acute abdominal pain in 46%.Twenty one (37%) of our patients had ovarian torsion. Four (7%) patients had benign tumors, and 8 (14%) had malignant tumors. There were no age differences between those with benign type (8.2±2.6years) and malignant tumors (6.1±5.3years) (P=0.683).

**Conclusion:**

Ovarian tumors are rare in children. Most are benign, in children presenting with acute abdominal pain, ovarian mass particularly neoplastic tumors should be suspected. An important proportion of these patients may require postoperative chemotherapy.

Ovarian tumors are uncommon in children, and the clinical presentation and pathology are different from adult patients (1). The cause of ovarian tumors in infant and children is unknown, however, ovarian neoplasms are estimated to occur in 2.6 cases per 100,000 girls per year and malignant ovarian neoplasms about 1%-2% of all children cancers (2-4). Pathologically, the nature of ovarian tumors ranges from benign cysts to highly aggressive malignant tumors, and with protean presentations (5). Ovarian masses are classified as non-neoplastic and neoplastic, and simple ovarian cysts are the most common mass in children (6). The most common ovarian masses in young adolescents are functional cysts, ovarian torsion, and benign neoplasms, and the most frequent malignant tumors of children is germ cell malignant tumor (7, 8). In children with acute abdominal pain, different clinical conditions like appendicitis, gastrointestinal gynecological disease may be considered which require extensive laboratory and imaging tests for definitive diagnosis. 

However, in patients requiring emergency operation, presumptive preoperative diagnosis is helpful for surgical or postoperative management. Data in this context particularly in children are scarce. The present study was designed to determine the cause of ovarian mass in children presented to Mofid Children’s Hospital from 2002-2012.

## Methods

Fifty-seven children with ovarian mass confirmed by ultrasonography and CT scan admitted to Mofid Children's Hospital from March 10, 2002 to April 2012 were studied. All cases with ovarian samples submitted for pathologic review were included for further evaluation data. Data regarding demographic data, clinical presentation, treatment, tumor histology, and outcome were collected from medical records. This retrospective analysis was performed with the approval of the hospital institutional review board (IRB). All data were recorded and evaluated using SPSS software Version 18.

## Results

**Fig 1:**
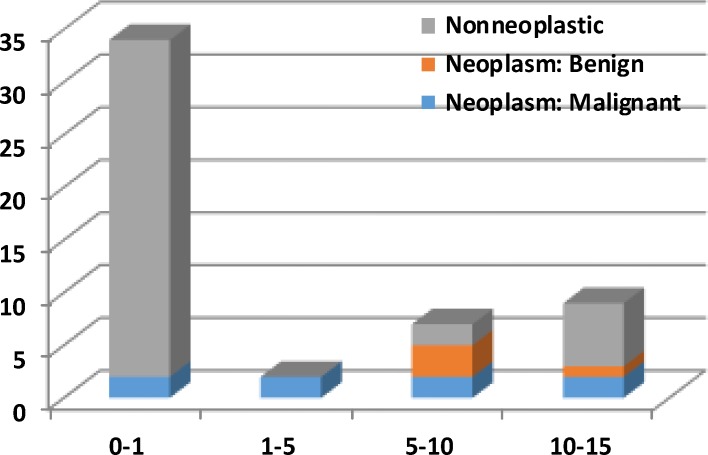
Distribution of age and type of pathology in our patients

The mean age of the study patients was 40.2±57 months, (range 1 day to 15 years). Eight (14%) girls had no symptoms on admission. The presenting symptoms were acute abdominal pain in 26 (45.6%), palpable abdominal mass 20 (35%), fever 3 (5.3%), nausea and vomiting 3 (5.3%) patients. Laboratory findings showed leukocytosis in 42.1% and anemia in 8.8% patients. The patients underwent different types of ovarian surgeries included; Salpingo-oophorectomy in 24 (42%) cases, oophorectomy in 10 (17.5%), ovarian cystectomy in 21 (36.8%), and ovarian biopsies in 2 (3.5%) patients. The total number of mass was 64 in which 50 children had unilateral ovarian masses; right side 49.1% and 38.6% in left side, and 12.3% had bilateral mass respectively. Intra-operative findings were as followes: torsion 32.8%, hemorrhagis 12.5%, and necrosis in 14.1%. Pathology types in our patients were seen in table 1, and the distribution of age and type of pathology in our patients was shown in figure 1. We observed 2 (3.5%) patients with seeding of tumor cells in a pathology report (1 case of germ cell tumor and the other is teratoma). 

The mean age of patients with ovarian cyst, benign and malignant tumors were 2.2±4.6, 8.2±2.6 and 6.1±5.3 years, respectively. There was a statistical significant difference between the mean of age in cases with ovarian cyst and benign (P=0.019) and malignant tumors (P=0.035), but there was no mean age difference between benign and malignant tumors in our study patients (P=0.683). 

All malignant masses received chemotherapy performed by a pediatric oncologist.

**Table 1:** The type of pathology series

**N (%)**	**Pathology**
40 (76.9) 7 (13.5) 27 (51.9) 6 (11.5)	**Nonneoplastic** Simple cyst Follicular cyst Corpus luteal cyst
4 (7.7) 4 (7.7)	**Neoplastic: Benign** Mature teratoma
8 (15.4) 2 (3.8) 1 (1.9) 3 (5.3) 2 (3.8)	**Neoplastic: Potential Malignant** Yolk sac tumor Juvenile granulosa cell tumor Immature teratoma Germ cell tumor

The mean size of patient masses with ovarian cyst, benign and malignant tumors were 60.6±21.4, 104.3±53.6 and 75.9±39.6 centimeters, respectively. There was statistical significant difference between the average mass size in cases with ovarian cyst and benign tumors (P=0.006), but there was no difference in the mean size of masses in patients with malignant tumors, benign tumors (P=0.373) and ovarian cyst (P=0.158) in our study patients.

**Table 2:** Comparison of the type of pathology series

**Pathology**	**Zhang M (13)** **n=521**	**Cass DL (2)** **n=106**	**Liu H (7)** **n=214**	**Our study** **n=52**
Non-neoplastic	92 (17.7%)	49 (46.2%)	78 (36.5%)	40 (76.9%)
Neoplastic: Benign	382 (73.3%)	47 (44.4%)	116 (54.2%)	4 (7.7%)
Neoplastic: Malignant	47 (9%)	10 (9.4%)	20 (9.3%)	8 (15.4%)

## Discussion

Ovarian tissue arises from the mesenchyme of the urogenital ridge, the germinal epithelium that covers the urogenital ridge, the germ cells which arise in the yolk sac, and follicles in the ovary develop from all three of these embryonic cell lines (9). Ovarian pathology is rare in childhood, and surgeries are uncommon (2). Ovarian masses are rare in this age, but they are the most common genital neoplasms occurring in these ages (10, 11). 

Ovarian masses may represent physiologic cyst, benign type of neoplasm, or malignant neoplasms. Presenting symptoms may be pain or asymptomatic mass. Pelvic sonography is a very useful tool in the diagnosis of ovarian cyst, and torsion in children (12). 

Acute abdominal pain (47%) was the most common presenting symptom in our study, but in Zhang et al.’s study series (12) it was 39.5%, and in Ammor et al.’s study (13) it was 55%. We found 37% ovarian torsion in our series, but in Cass et al.’s study (2) 42% had ovarian torsion. 87.7% of our ovarian mass was unilateral, only 12.3% were bilateral, which was comparable to Bhattacharyya et al.’s study (14), but in McCarville et al.’s study series, bilateral ovarian mass was 33%. Most of non-neoplastic pathology in our study was follicular cyst (51.9%), benign neoplastic was mature teratoma (7.7%), and malignant neoplastic was germ cell tumor (5.3%). In our previous study, 32 out of 44 patients with germ cell tumor had ovarian mass (15). In a study by Cass et al. (2), most common non-neoplastic lesion was ovarian torsion (15.1%), and benign neoplastic was mature teratoma (36.9%), and malignant neoplastic was dysgerminoma (3.6%), and in Morowitz et al.’ series study (18), non- neoplastic was 51%, and neoplastic mass mostly was germ cell (33%) (16). Neonatal ovarian masses are usually cystic and mostly are resolved spontaneously within 8 to12 months because of withdrawal of maternal hormonal stimulation (17). 

Detorsion and preservation of all ovaries, even in necrotic cases has been recently recommended, also, aspiration of simple benign appearing cyst or contralateral ovary may be helpful (18). In our study, benign mature teratoma was 7.5% that is treated by either oophorectomy or salpingo-oophorectomy. In recent study, malignant germ cell tumor rate has been 5.3%, which was treated by salpingo- oophorectomy (19), but in Dessouky et al.’s study (20) series, this rate was 24%. Laparoscopic surgery can be performed in all cystic lesions for biopsy/aspiration/detorsion/excision, as it is a safe operative approach with good cosmetic results in children and young girls (21).

In conclusion ovarian tumors are rare in children. Abdominal pain was the most common complaint. The most masses are non-neoplastic (cystic). Surgical treatment is conservative for both benign and malignant lesions; however, postoperative chemotherapy is needed in most malignant lesions.

**Limitation: **We had limitations in tumor marker evaluation and small sample size.

## Notes

**Funding:** This study was financially supported by the Office of the Vice Chancellery for Medical Research Development Center (MRDC) of Mofid Children's Hospital.
